# New thiadiazole modified chitosan derivative to control the growth of human pathogenic microbes and cancer cell lines

**DOI:** 10.1038/s41598-022-25772-4

**Published:** 2022-12-11

**Authors:** Ahmed G. Ibrahim, Amr Fouda, Walid E. Elgammal, Ahmed M. Eid, Mohamed M. Elsenety, Ahmad E. Mohamed, Saber M. Hassan

**Affiliations:** 1 Department of Chemistry, Faculty of Science, Al-Azhar University, Nasr City, Cairo Egypt; 2 Department of Botany and Microbiology, Faculty of Science, Al-Azhar University, Nasr City, Cairo Egypt

**Keywords:** Biotechnology, Cancer, Drug discovery, Microbiology, Chemistry, Engineering, Materials science

## Abstract

The emergence of multidrug-resistant microbes and the propagation of cancer cells are global health issues. The unique properties of chitosan and its derivatives make it an important candidate for therapeutic applications. Herein, a new thiadiazole derivative, 4-((5-(butylthio)-1,3,4-thiadiazol-2-yl) amino)-4-oxo butanoic acid (BuTD-COOH) was synthesized and used to modify the chitosan through amide linkages, forming a new thiadiazole chitosan derivative (BuTD-CH). The formation of thiadiazole and the chitosan derivative was confirmed by FT-IR, ^1^H/^13^C-NMR, GC–MS, TGA, Elemental analysis, and XPS. The BuTD-CH showed a high antimicrobial effect against human pathogens *Escherichia coli*, *Pseudomonas aeruginosa*, *Bacillus subtilis*, *Staphylococcus aureus*, and *Candida albicans* with low MIC values of 25–50 μg ml^−1^ compared to unmodified chitosan. The in-vitro cytotoxicity of BuTD-CH was evaluated against two cancer cell lines (MCF-7 and HepG2) and one normal cell (HFB4) using the MTT method. The newly synthesized derivatives showed high efficacy against cancerous cells and targeted them at low concentrations (IC_50_ was 178.9 ± 9.1 and 147.8 ± 10.5 μg ml^−1^ for MCF-7 and HepG2, respectively) compared with normal HFB4 cells (IC_50_ was 335.7 ± 11.4 μg ml^−1^). Thus, low concentrations of newly synthesized BuTD-CH could be safely used as an antimicrobial and pharmacological agent for inhibiting the growth of human pathogenic microbes and hepatocellular and adenocarcinoma therapy.

## Introduction

Cancer is one of the global health issues that have yet to be addressed^1^. Fortunately, the exclusive properties of chitosan and its derivatives in terms of biodegradability, non-immunogenicity, and biocompatibility make it an important candidate for therapeutic applications. Moreover, chitosan cross-linked grafts manifested antimicrobial activity as well as anticancer efficacy with minimal toxicity on normal cells^2^. Chitosan is one of the natural polymers that can be employed in many applications due to the presence of functional hydroxyl (‒OH) and amino (‒NH_2_) groups. These groups provide active centers for conducting many reactions on them and provide the synthesis of many chitosan derivatives with many compounds that can have coordination^3,4^, antibacterial^5–7^, antifungal^8^, antioxidant^9,10^, antiviral properties^11^, and others. In addition, chitosan has economic attractiveness as it can be easily obtained from chitin by deacetylation. Chitin is one of the most important natural polymers after cellulose. Chitin is found in fungal cell walls as well as in the shells of crustaceans such as crabs, shrimp, and lobsters^12^. The process of deacetylation of chitin to prepare chitosan occurs at high temperatures and in an alkaline medium, where the amino groups in position number two are liberated due to hydrolysis^13^. Chitosan has unique properties such as biodegradability, biocompatibility, non-toxicity, and its activity against bacteria. All these properties made it widely used in the field of biomedicine, pharmaceuticals, and environmental pollutants treatment, for example, tissue engineering, gene delivery, and drug delivery^7,14–16^.

Many researchers have prepared chitosan derivatives by reacting its functional groups with heterocyclic compounds containing nitrogen^14,17–19^, sulfur^20–22^, or both^4,5,18^.

Heterocyclic compounds have attracted the attention of many researchers, as many of them are active against bacteria^23^, fungi^24^, inflammations^25,26^, and cancer^27,28^. Thiadiazole is one of the important heterocyclic compounds containing nitrogen and sulfur. 1,3,4- Thiadiazoles are very interesting heterocycles compounds because of the contained (–N=N–C–S) group that can be constituted H-bonding interactions with appropriate receptor domains and exhibit varied biological applications^29^. So, possessing this property, 1,3,4-thiadiazole derivatives are utilized broadly in pharmaceutical, agricultural, coordination chemistry, and materials chemistry^30,31^. Heterocyclic compounds incorporated thiadiazole nucleus demonstrated a wide range of biological activities such as antioxidant^32^, cytotoxic^33^, antibacterial^34^, antipsychotic^35^, anti-inflammatory^36^, analgesic^37^, antiviral^38^, antimicrobial^39^, antihypertensive^40^, antileishmanial^41^, and antihistamine^42^. Mainly, the thiadiazole nucleus has been involved in different medicinal drugs such as the Azetepa (DNA alkylating agent)^43^, acetazolamide, and methazolamide (carbonic anhydrase inhibitors for glaucoma treatment), sulfamethizole, cefazedone, cefazolin, and ceftezole (as antibacterial drugs)^44^.

Therefore, the main hypothesis of the current study was the synthesis of thiadiazol derivative for the first time and its use to functionalize chitosan in an attempt to get efficient antimicrobial-modified chitosan with high cytotoxic efficiency. To achieve this hypothesis, a new thiadiazole derivative, 4-((5-(butylthio)-1,3,4-thiadiazol-2-yl) amino)-4-oxo butanoic acid (BuTD-COOH), was synthesized and used to functionalize chitosan through amide linkages forming a new thiadiazole chitosan derivative (BuTD-CH). The new derivative was characterized by FT-IR, ^1^H/^13^C-NMR, GC–MS, TGA, elemental analysis, and XPS. The biological activity includes antimicrobial against pathogenic clinical isolates and in-vitro cytotoxic efficacy against two cancerous cell lines (MCF-7 and HepG2) and one normal cell line (HFB4) were investigated and compared with the activity of unmodified chitosan.

## Results and discussion

### Modification of chitosan

In this work, chitosan as a natural polysaccharide was modified by a new thiadiazole derivative, 4-((5-(butylthio)-1,3,4-thiadiazol-2-yl) amino)-4-oxo butanoic acid (BuTD-COOH), by the formation of amide linkages between the carboxylic group of the thiadiazole derivative and the amino groups of chitosan. The thiadiazole derivative was synthesized through three successive steps. First, 5-amino-1,3,4-thiadiazole-2-thiol (TD-NH_2_) was synthesized by reacting thiosemicarbazide with carbon disulfide as reported previously^45^. Second, TD-NH_2_ was reacted with butyl iodide as an alkyl halide to get BuTD-NH_2_ in the presence of KOH as an HI scavenger. Finally, the primary amine (BuTD-NH_2_) as a nucleophile was reacted with an acid anhydride (succinic anhydride) to obtain the carboxylic derivative BuTD-COOH.

### FTIR spectroscopy

**Figure 1:**
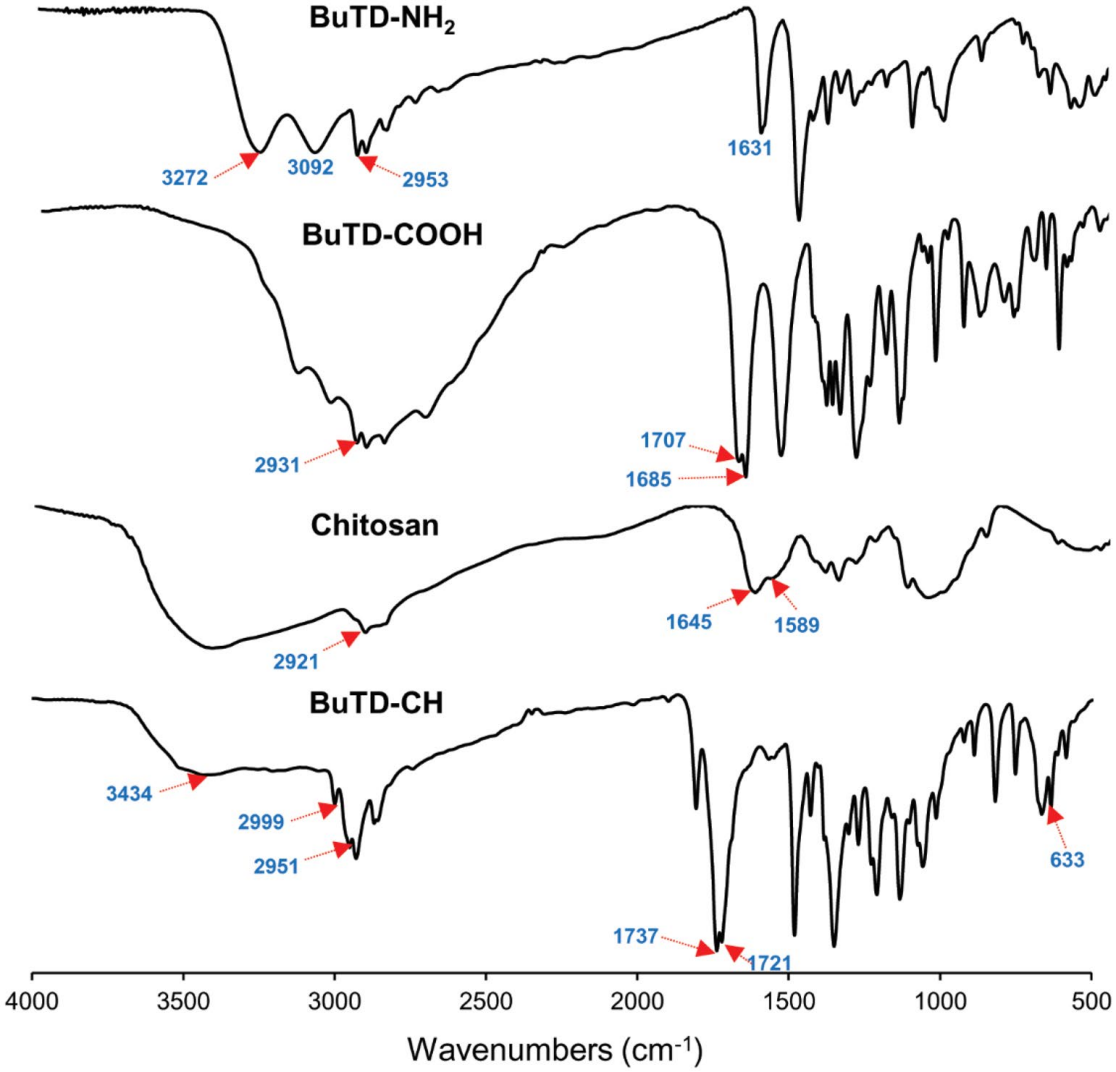
FTIR spectrum of BuTD-NH_2_, BuTD–COOH, Chitosan, and BuTD–CH.

In the FTIR spectrum of the BuTD–NH_2_ derivative (Fig. 1), the characteristic broad bands at 3272 cm^−1^ and 3092 cm^−1^ are ascribed to the stretching vibrations of the amino group ʋ(NH_2_), and the stretching vibration absorption band observed at 2953 cm^−1^ is assigned to aliphatic ʋ(CH). Furthermore, an absorption band around 1631 cm^−1^, due to azomethine group ʋ(C=N), was observed. The FTIR spectra of the carboxylic intermediate (BuTD-COOH) revealed the absence of the NH_2_ group′s absorption band, as well as peaks at 3274 cm^−1^ stretching frequency, indicating the presence of hydroxyl group ʋ(OH), including a peak at 3154 cm^−1^ attributed to ʋ(–NH). In addition, there were stretching bands at 2931 cm^−1^ for C–H aliphatic, 1707 cm^−1^ for the carbonyl group of a carboxylic acid ʋ(COOH), 1685 cm^−1^ for the carbonyl group of ʋ(NH–C=O), and 1569 cm^−1^ attributed to the olefinic group ʋ(C=C), respectively. In the infrared spectrum of chitosan, the stretching vibrations for both O–H and NH_2_ (overlapped) were observed in the region 3331–3291 cm^−1^. The small broad peaks at 2921 cm^−1^ and 2877 cm^−1^ were related to the stretching vibrations of the C–H (symmetrical and asymmetrical) in CH_2_OH and the pyranose rings. The presence of the broadband at around 1645 cm^−1^ was due to the stretching vibrations of the C=O (Amide I) and the small broad shoulder at 1589 cm^−1^ was related to the bending vibrations of the NH in NH_2_ (Amide II)^46^. Moreover, the characteristic bands observed at 1423 cm^−1^, 1375 cm^−1^, and 1262 cm^−1^ were associated with the bending vibrations of OH in CH_2_OH, deformation, wagging, and twisting vibrations of the CH_2_ in CH_2_OH and pyranose rings, and stretching vibrations of CH_3_ symmetrical in the acetyl-amide groups. The peak at 1325 cm^−1^ was assigned to the C–N stretching of (amide III), as well as the characteristic bands at 1154 cm^−1^, 1066 cm^−1^, and 1028 cm^−1^ were attributed to the stretching vibration of C–O in CH_2_OH, the symmetric and asymmetric stretching vibrations of both the C–O–C bridge and the C–O in the chitosan, respectively, and the small peak at 896 cm^−1^ was ascribed to the skeletal vibrations of chitosan^47^.

In comparison to the FTIR spectrum of chitosan, the FTIR spectrum of BuTD-CH showed a shifting of the band 3331–3434 cm^−1^ with an obvious increase in the broadening due to overlapping of NH groups and OH groups. New absorption bands in the FTIR spectrum of BuTD–CH have appeared. The bands appeared at 2999 cm^−1^ and 2951 cm^−1^ ascribing to the CH_2_–vibrations of the succinyl chain (–CH_2_–CH_2_–). The carbonyl groups shifting to 1721 cm^−1^ and 1737 cm^−1^, the disappearance of the characteristic absorbance of –NH_2_ at 1589 cm^−1^, and the appearance of new bands at 633 cm^−1^ ʋ (C–S)^48^ evidenced the introduction of the thiadiazole derivative to the chitosan structure.

### ^1^H,^***13***^C- NMR spectral and mass spectrum

^1^H-NMR(CDCl_3_,500 MHz) (Figure S1a,b) of the BuTD–NH_2_ intermediate **(**δ ppm) = 5.91 (s,2H,N**H**_**2**_, exchangeable by D_2_O), 3.09(t, *J* = 7.2 Hz, 2H, S**CH**_**2**_ C_3_H_7_), 1.68 (m, *J* = 7.6 Hz, 2H,SCH_2_**CH**_**2**_ C_2_H_5_), 1.42 (m, *J* = 7.6 Hz**,** 2H, S(CH_2_)_2_**CH**_**2**_CH_3_) and 0.90 (t, *J* = 7.2 Hz, 3H, S(CH_2_)_2_CH_2_**CH**_**3**_). Moreover,^13^C-NMR (CDCl_3_, 125 MHz) (Figure S1c) appeared the presence of signals (δ ppm) = 169.54 (S–**C** = NS),154.52 (N–**C** = NS), 34.94(S**C**H_2_C_3_H_7_),31.49 (SCH_2_ **C**H_2_C_2_H_5_), 21.83 (SC_2_H_4_**C**H_2_CH_3_) and 13.64 (SC_3_H_6_**C**H_3_) and the mass spectrum (Figure S1d), has the molecular ion m/z = 189 (3.65%), confirming its presumed structure. Also, ^1^H–NMR (DMSO-d_6_,500 MHz) (Figure S2a,b) of BuTD–COOH revealed the signals **(**δ ppm) = 12.60 (s, 1H, COO**H**, exchangeable by D_2_O), 12.22 (s, 1H, CON**H**, exchangeable by D_2_O), 3.16 (t, 2H, (t, *J* = 7.2 Hz, 2H,S**CH**_**2**_C_3_H_7_), 2.65 (t, *J* = 6.8 Hz, 2H, CH_2_–acidic), 2.52 (t, *J* = 6.4 Hz, 2H,CH_2_-amidic), 1.61(m, *J* = 7.6 Hz, 2H,SCH_2_**CH**_**2**_C_2_H_5_), 1.36 (m, *J* = 7.2 Hz, 2H, S(CH_2_)_2_ **CH**_**2**_CH_3_) and 0.85(t, *J* = 7.2 Hz, 3H, S(CH_2_)_2_CH_2_**CH**_**3**_). ^13^C-NMR (DMSO-d_6_, 126 MHz) (Figure S2c) (δ ppm) = 173.93 (**C**OOH), 171.27 (NH–**C**=O), 159.12(S–**C**=NS), 159.05(N–**C**=NS), 33.92(S**C**H_2_C_3_H_7_), 31.60(SCH_2_**C**H_2_C_2_H_5_), 30.38(CH_2_-acidic), 28.87(CH_2_-amidic), 21.67 S(CH_2_)_2_**CH**_**2**_CH_3_), 13.92 S(CH_2_)_2_CH_2_**CH**_**3**_). In addition, the mass spectrum of the compound (BuTD-COOH) (Figure S2d) revealed a molecular ion peak at m/e = 289.37 (2.4%), which agreed with the suggested structure.

**Figure 2:**
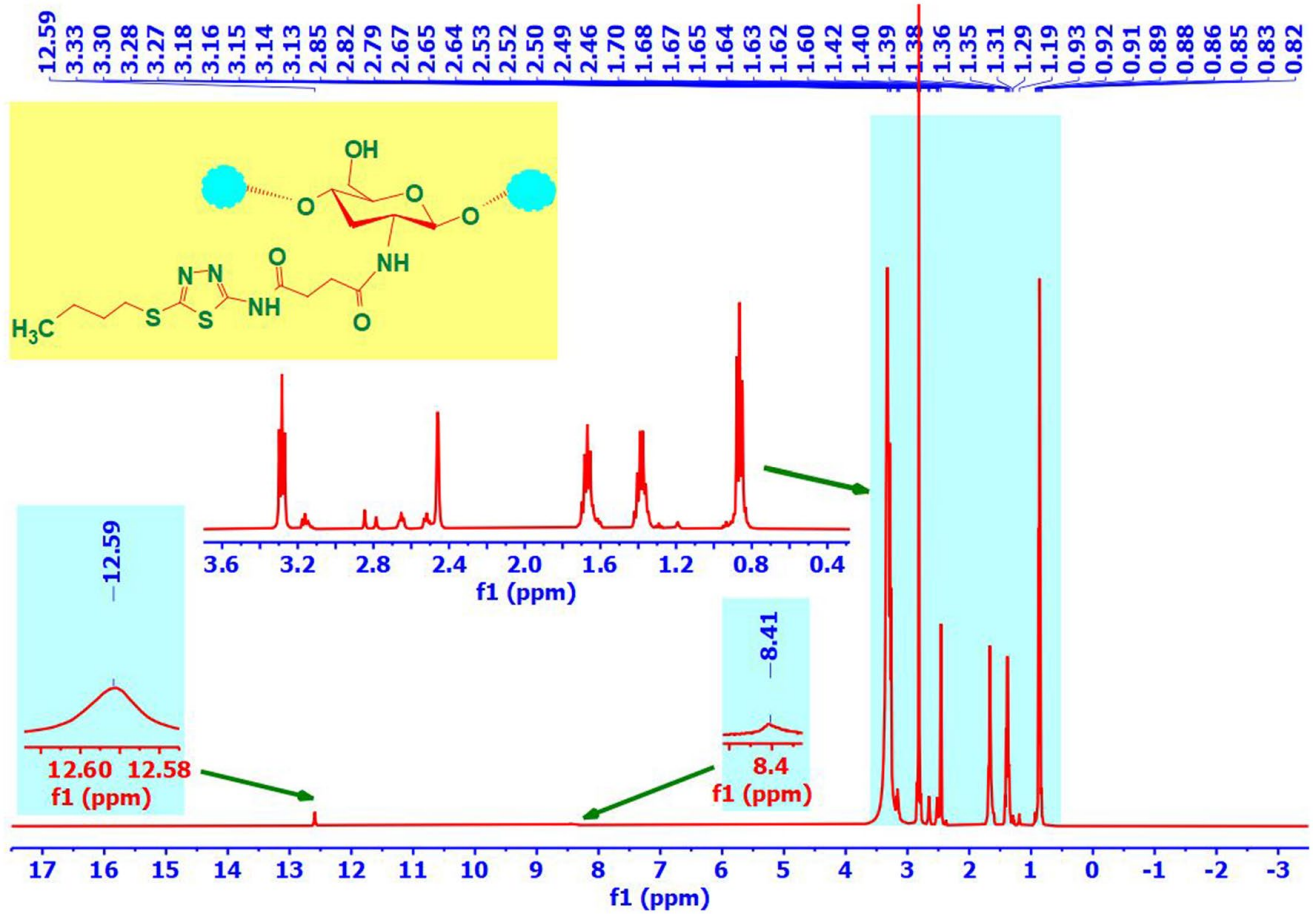
^1^H-NMR (DMSO-D_6_) analysis of BuTD–CH.

The ^1^H-NMR(DMSO-d_6_,500 MHz) spectrum of BuTD-CH (Fig. 2) showed the characteristic peaks of chitosan in addition to significantly different peaks, which confirmed the incorporation of the thiadiazole derivative into the chitosan skeleton. The chemical shift of the protons of the glucosamine group was observed at 3.33–5.71 ppm and the weak signal at 2.46 ppm due to the three protons of the methyl of the CH_3_CO–NH group of chitosan^49,50^. New signals **(**δ ppm) were observed in the spectrum of BuTD-CH at 12.59 (s, 1H, NH) referring to SCNH–CO of thiadiazole derivative and 8.43 (s, 1H, NH) referring to the amide linkage. Signals observed at 3.28 ppm, 1.67 ppm, 1.38 ppm, and 0.86 ppm are attributed to three methylene and methyl of an n-butyl group. Signals that appeared at 2.65 ppm and 2.52 ppm originated from methylene groups of the succinic unit. All the above-mentioned new peaks confirmed the successful synthesis of the new chitosan derivative (BuTD-CH).

### Thermal gravimetric analysis (TGA)

**Figure 3:**
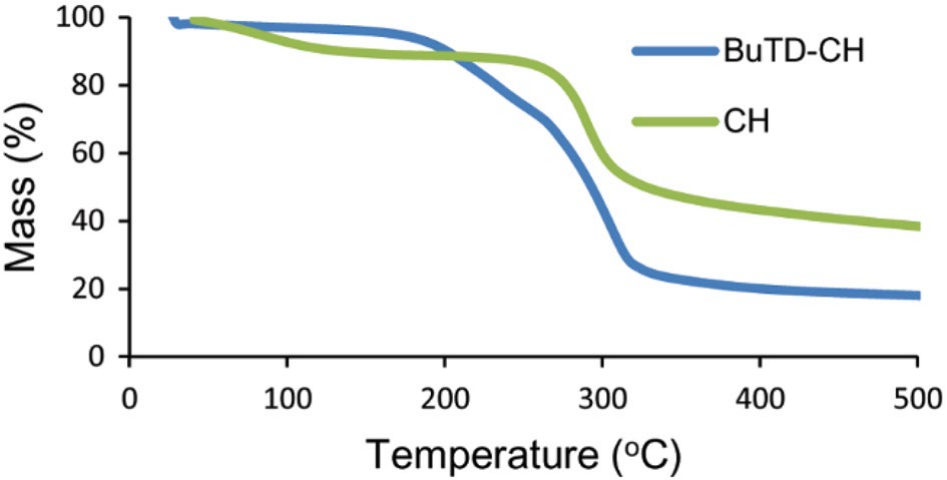
TGA curve of chitosan (CH) and the modified chitosan (BuTD–CH).

Using TGA analysis, the thermal behavior of the new chitosan derivative was investigated and compared with that of pure chitosan. The results were presented in Table 1 in terms of mass loss (%) at 200, 400, and 600 °C, onset decomposition temperature (T_o_), the temperature of 50% weight loss (T_50_), and temperature of maximum decomposition rate (T_max_), and the results of mass loss (%) were plotted against temperature (°C), as shown in Fig. 3. Figure 3 shows that chitosan lost 10.55% of its mass at a temperature of 129.26 °C, while the new chitosan derivative (BuTD-CH) lost only 3.73% of its weight at the same temperature. This is due to the decrease in the number of amino groups in the modified chitosan, where amino groups are linked to water molecules through hydrogen bonds, and the loss in sample mass at this stage represents the desorption of water molecules. Figure 3 shows that chitosan decomposes in one stage, starting at a temperature of 266.08 °C (12.30% mass loss), while the BuTD–CH decomposes in two stages, the first at 185.27 °C (4.79% mass loss) as a result of the degradation of the heterocyclic compound, and the second at 271.12 °C (32.55% mass loss) as a result of the decomposition of the chitosan chain. From the results presented in Table 1, it is clear that the thermal stability of chitosan decreases after the introduction of the heterocyclic compound into its composition.

**Table 1:** Data of thermal gravimetric analysis for chitosan (CH) and newly modified chitosan (BuTD–CH).

	T_o_ (°C)	Mass loss (%) at temperature (°C)			T_50_ (°C)	T_max_ (°C)
		200	400	600		
CH	266.08	11.387	56.796	65.256	327.4	290.07
BuTD–CH	185.27	9.677	79.972	83.913	293.1	234.60 (1st), 307.40 (2nd)

### Elemental analysis

The elemental analysis of unmodified chitosan and modified chitosan was scanned, and the results were found to be 36.38%(C), 5.56%(H), and 6.05%(N) for unmodified chitosan and 35.54%(C), 3.7%(H), 11.01%(N), and 37.51%(S) for BuTD-CH. The elemental analysis data was used to predict the degree of substitution (DS) from the mathematical relationship shown below^51^.

1 $$DS\left( \% \right) = \frac{{\alpha X - {\text{Y}}}}{{\text{b}}} \times 100$$

where *X* and *Y* are the carbon/nitrogen ratios of BuTD-CH and chitosan, respectively. α and b are the numbers of nitrogen and carbon elements, respectively, added to chitosan after modification with the BuTD nucleus. Decreasing C% and increasing N% indicate the modification success of chitosan with a degree of substitution of 42%.

### X-ray photoelectron spectroscopy

**Figure 4:**
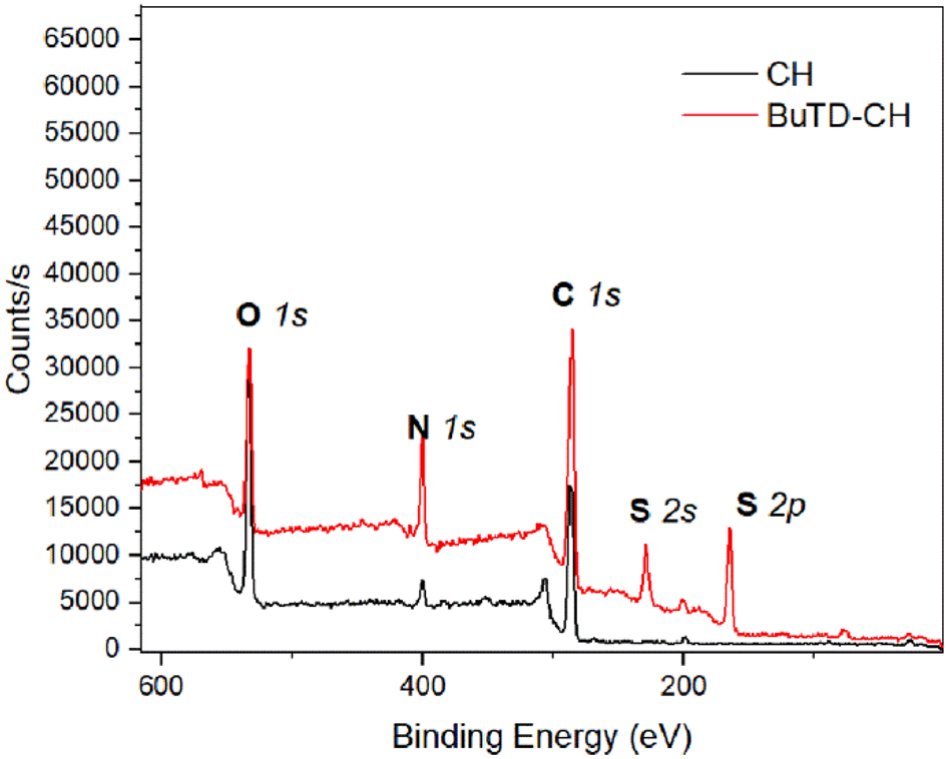
Full scans of XPS spectra of chitosan (CH) and its BuTD-CH derivative.

XPS examinations are a significant tool for surface analysis, due to their ability to distinguish between different chemical forms of particular atoms based on their oxidation states^52^. Figure 4 represents the full XPS spectra of chitosan and its BuTD-CH derivative.

It is clear that the sulphur signals at roughly 226 eV and 166 eV, which are assigned to S *2 s* and S *2p*, respectively, are present in the spectrum of the chitosan derivative of BuTD-CH, as opposed to the chitosan spectrum, which correlates to the presence of sulphur atoms in chitosan's derivatives^53^. Moreover, the carbon (C *1 s*), oxygen (O *1 s*), and nitrogen (N *1 s*) signals of the BuTD–CH were slightly displaced combined with increased intensity compared to the CH peaks, which shows the rising quantity of deacetylation of chitosan and the formation of the new derivatives^54^.

**Figure 5:**
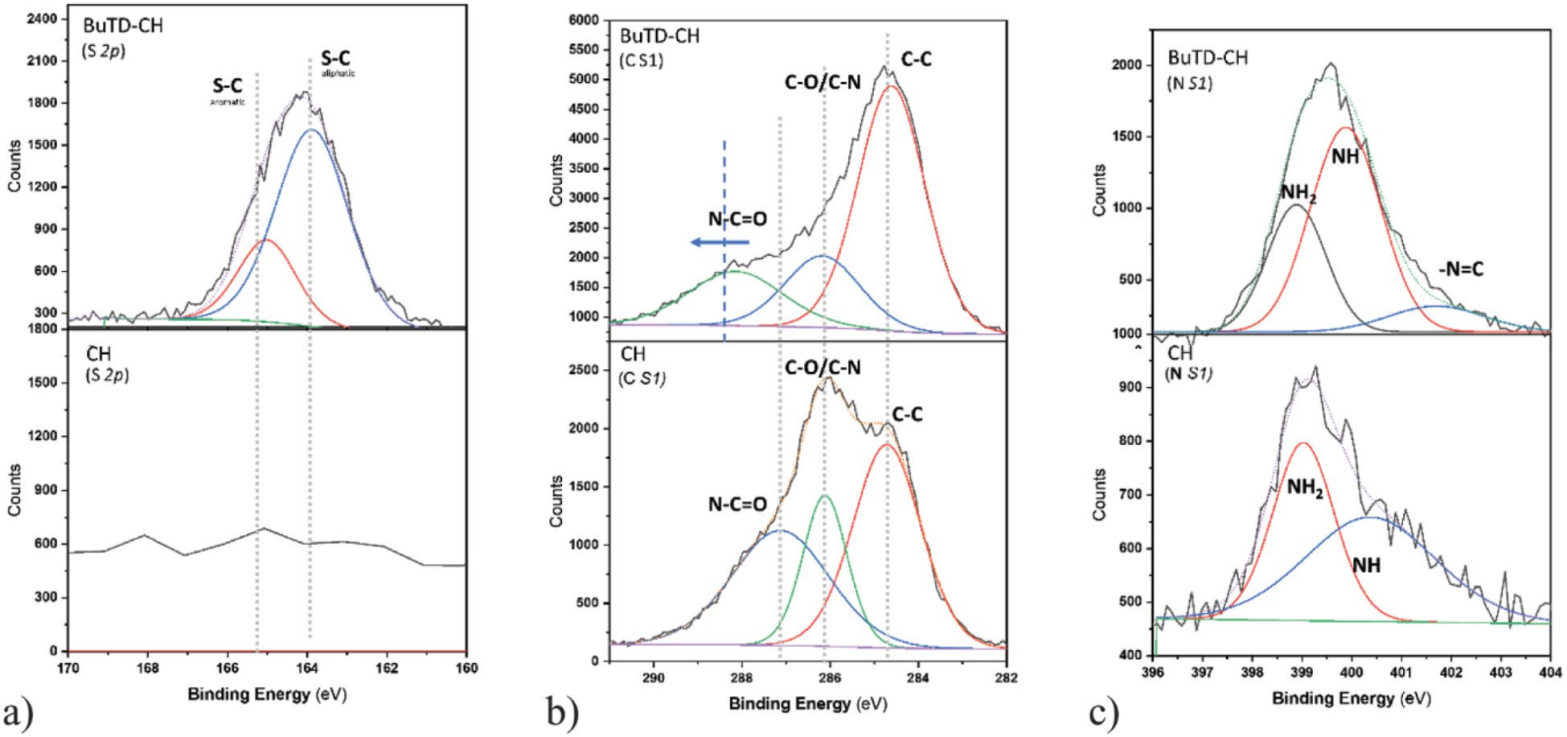
XPS spectra of chitosan (CH) and its derivative of BuTD–CH, and bending energy of Sulfur S *2p* (**a**); Carbon C *1 s* (**b**); Nitrogen N *1 s* (**c**).

Furthermore, the S *2p* peaks at 163.9 and 165.3 eV (Fig. 5a) were associated with S-C in the aliphatic and aromatic groups, respectively^55^. Meanwhile, significant positive correlations were found at C *1 s* and N *1 s* peaks. The XPS spectrum of CH for C *S1* presents three deconvoluted peaks at 284.7, 286.1, and 287.1 eV which are attributed to C–C, C–N/C–O, and N–C=O respectively^56^, as shown in Fig. 5b. As expected, the significant shift of the peak at 284.7 eV for BuTD-CH, toward higher binding energy as a result of the substituted acetyl group with a different terminal chain of the polymer was also significant. However, Fig. 5c shows the corresponding peaks of N *1 s* of chitosan compound at 399 and 400.5 eV which are attributed to NH_2_ and NH, respectively. New peaks at ~ 402 eV related to –N=C occurred with a gradual shift, after substitution for chitosan’s derivatives of BuTD-CH. No noteworthy differences were found in the spectra of O *1S* for chitosan or its derivatives. Overall, the results show a significant relationship between the formation of BuTD-CH polymers.

### Antimicrobial activity

**Figure 6:**
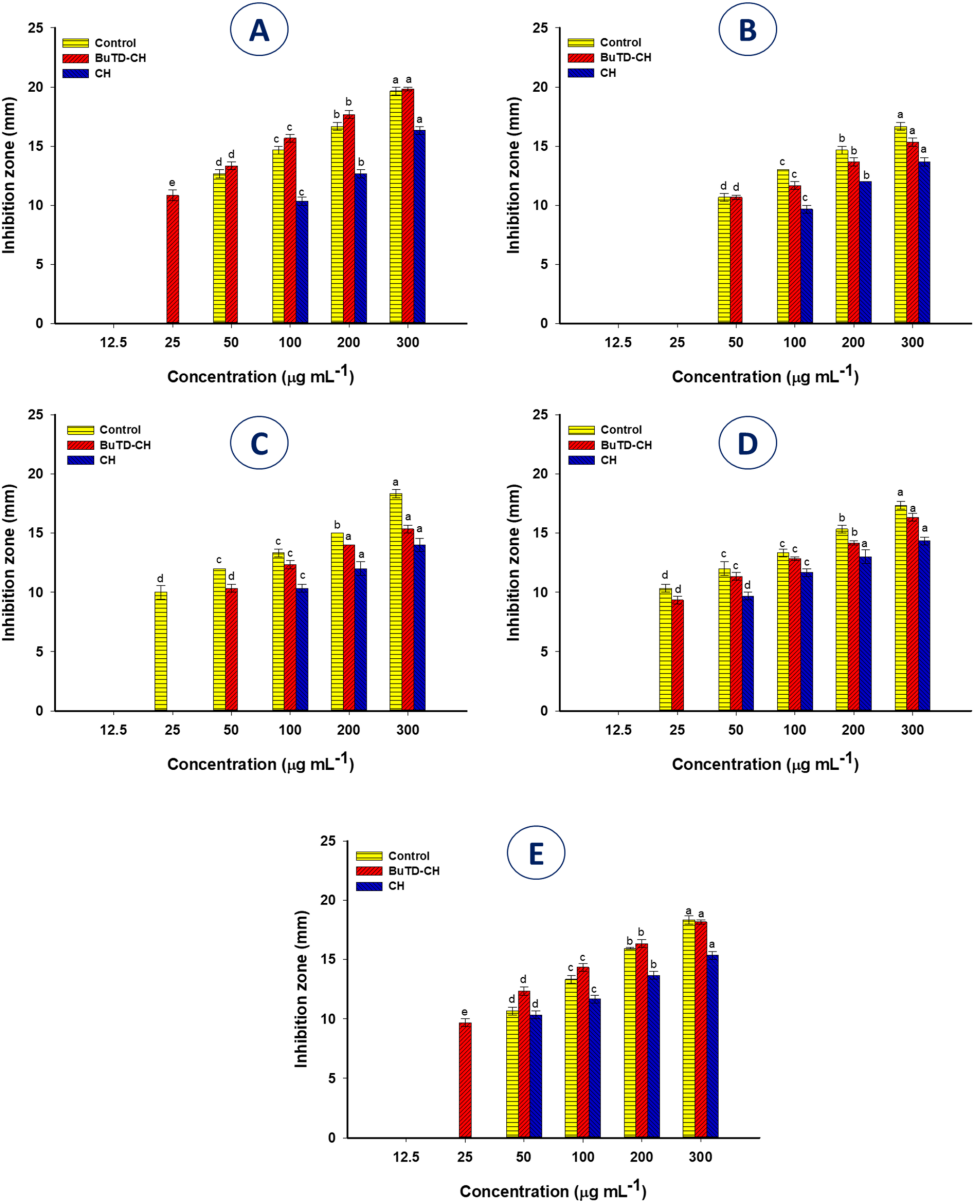
The antimicrobial activity of new active butylated chitosan compared with unmodified chitosan against different pathogenic Gram-positive bacteria, *B. subtilis* (**A**) and *S. aureus* (**B**), Gram-negative bacteria, *P. aeruginosa* (**C**) and *E. coli* (**D**), and unicellular fungi *C. albicans* (**E**). Data are statistically analyzed at *p* ≤ 0.05 using Tukey’s test (n = 3, ± SD). Bars with the same letters at different concentrations are means that the data are not significantly different.

The prevalence of multidrug-resistant bacterial pathogens has increased the global expansion of life-threatening infections, which necessitates the production and development of pioneering antimicrobials, essentially materials derived from natural products such as functionalized chitosan, which has recently shown efficacy as a broad-spectrum antibiotic^57,58^. Herein, the agar well diffusion screening was applied to evaluate the antimicrobial potential and minimum inhibitory concentrations of the butylated thiadiazole modified (BuTD-CH) and unmodified chitosan compared with the positive and negative control^59^. The test was run against a consortium of distinct clinical pathogens comprising Gram-negative bacteria (*Escherichia coli* and *Pseudomonas aeruginosa*), Gram-positive bacteria (*Staphylococcus aureus* and *Bacillus subtilis*), and the model fungus (*Candida albicans*). Our results showed that DMSO has always been proven as a safe solvent^60^, so we used it in this experiment as a negative control and it did not show any interference with the growth of any of the tested microbes. At the lowest concentration (25 μg ml^-1^), the BuTD-CH, unmodified chitosan, and the selected positive-control antibiotics effectively inhibited the growth of the tested pathogens with variable efficacy in a dose-dependent manner. Interestingly, the BuTD–CH was more active against all tested organisms at low concentrations compared to unmodified chitosan. For instance, 25 μg ml^−1^ of the BuTD–CH inhibited the growth of *B*. *subtilis* and *C*. *albicans*; recording inhibition zones (ZOI) 10.83 ± 0.76 and 9.66 ± 0.57 mm respectively, while the application of the same concentration of unmodified chitosan, penicillin G, and the fungicidal ketoconazole did not (Fig. 6A,E). Chitosan is one of the leading biopolymers for its non-toxicity, biocompatibility, accessibility, biodegradability, and respectable antibacterial properties^61,62^. Moreover, modifying the structure of chitosan with different types of functional groups with cationic properties may increase its reactivity^63^. This may increase its solubility and modify the pH value to be in the physiological range, which improves its properties as an antibiotic. The efficacy of N-butyl chitosan derivatives was demonstrated as a broad-spectrum antibacterial agent against *Staphylococcus aureus*, *Escherichia coli*, and *Pseudomonas aeruginosa* using concentrations of 46, 128, and 128 μg ml^−1^, respectively^64^.

When the maximum concentrations (300 μg ml^−1^) were tested, both modified chitosan and penicillin G manifested their maximum activity against *B*. *subtilis* with ZOIs of 19.83 ± 0.28 and 19.66 ± 0.57 mm, respectively. There is no significant difference between the inhibitory effects of both. Whereas, the clear zone formed against *B*. *subtilis* due to treatment with a maximum concentration of unmodified chitosan was 16.3 ± 0.6 mm. On the contrary, *S. aureus* showed the lowest sensitivity to the BuTD-CH derivative and penicillin G, recording ZOIs of 15.33 ± 0.57 and 16.66 ± 0.57 mm, respectively, with a significant difference between the inhibitory effects of both (*p* < 0.05) (Fig. 6 B) as compared to unmodified compound (ZOI is 13.7 ± 0.6 mm). This can be attributed to the low hydrophilicity of cell wall of Gram-positive bacteria compared to Gram-negative bacterial cell wall, which makes them less susceptible to the impact of chitosan^65^. By and large, the characteristic antibacterial properties of chitosan are affected by its structure. Chitosan compounds with a large molecular weight cannot penetrate the cell wall, but they change cell permeability and prevent the passage of nutrients and essential minerals. Furthermore, low molecular weight chitosan penetrates the cell to affect mitochondrial functions, protein synthesis, and RNA^66^.

Identically, Gram-negative bacteria showed variable sensitivity as 300 μg ml^–1^ of BuTD-CH derivative effectively suppressed the growth of *P. aeruginosa* and *E. coli* with ZOIs of 15.33 ± 0.57 and 16.33 ± 0.57 mm, respectively. While 300 μg ml^–1^ of each ciprofloxacin and chitosan derivative had comparable activity against *E. coli*. However, the potency of 300 μg ml^–1^ ciprofloxacin was significantly higher than that of modified chitosan against *P. aeruginosa* (18.33 ± 0.57 and 15.33 ± 0.57 mm, *P* < 0.05) (Fig. 6C,D). In contrast the unmodified chitosan exhibit inhibitory effects toward *P. aeruginosa* and *E. coli* with ZOIs of 14.0 ± 1.0 and 14.3 ± 0.6 mm respectively at a maximum concentration (300 μg ml^–1^) (Fig. 6C,D). Lipopolysaccharide as a component of the cell wall of Gram-negative bacteria increases the negative charges of the cell surface and thus enhances the binding of the cell to the cationic chitosan, especially at pH < 6.5^67^.

Interestingly, the antifungal properties of BuTD-CH were similar to the effect of ketoconazole, as there was no significant difference for the effect of 300 μg ml^–1^ of both against *C. albicans* with ZOIs of 18.16 ± 0.28 and 18.33 ± 0.57 mm, respectively compared to unmodified chitosan that displayed ZOI of 15.3 ± 0.6 mm. The antifungal potential of chitosan derivative could be attributed to the hyperpolarization of the plasma membrane of *C. albicans* resulting from its strong electrostatic binding to chitosan, which leads to the flowing out of negatively charged molecules in cells such as substrates for enzymatic reactions, nucleotides, and phosphates^68^.

The effectiveness of infection control depends on the treatment strategy chosen based on the dependable evaluation of MIC^69^. Our investigations revealed that the lowest MIC records 25 μg ml^–1^ of BuTD-CH were assigned against *B. subtilis*, *E. coli,* and *C. albicans* with ZOIs of 10.38 ± 0.76, 10.33 ± 0.57, and 9.66 ± 0.57 mm, respectively. The MIC value of modified chitosan increased to 50 μg ml^–1^ against *S. aureus* and *P. aeruginosa* with ZOIs of 10. 66 ± 0.28 and 10.33 ± 0.57 mm, respectively (Fig. 6). Whereas the MIC values of unmodified chitosan were increased to 100 μg ml^–1^ toward Gram-positive bacteria (*B. subtilis* and *S. aureus*), and *P. aeruginosa*, and 50 μg ml^–1^ against *E. coli,* and *C. albicans.* Similarly, the MIC values of chitosan and its Schiff base derivatives against *S. aureus*, *B. cereus, Salmonella* sp., *P. aeruginosa*, *E. coli,* and *C. albicans* were in the range of 25−100 μg ml^–1^ ^70^. The current examination revealed that the MIC value of ciprofloxacin was 25 μg ml^–1^ against Gram-negative tested pathogens, while the MIC values of penicillin G and ketoconazole against Gram-positive and fungal pathogens, respectively, were perceived at 50 μg ml^–1^. These findings are consistent with previous studies confirming that the elimination of fungal infections may require intensive doses, as they have a very solid cell wall containing chitin and glucan and is completely different from the bacterial cell wall^71,72^. Overall, it can be concluded that the antimicrobial activity of functionalized chitosan with thiadiazole derivative was increased compared to unmodified compound.

### In-vitro cytotoxicity assay

**Figure 7:**
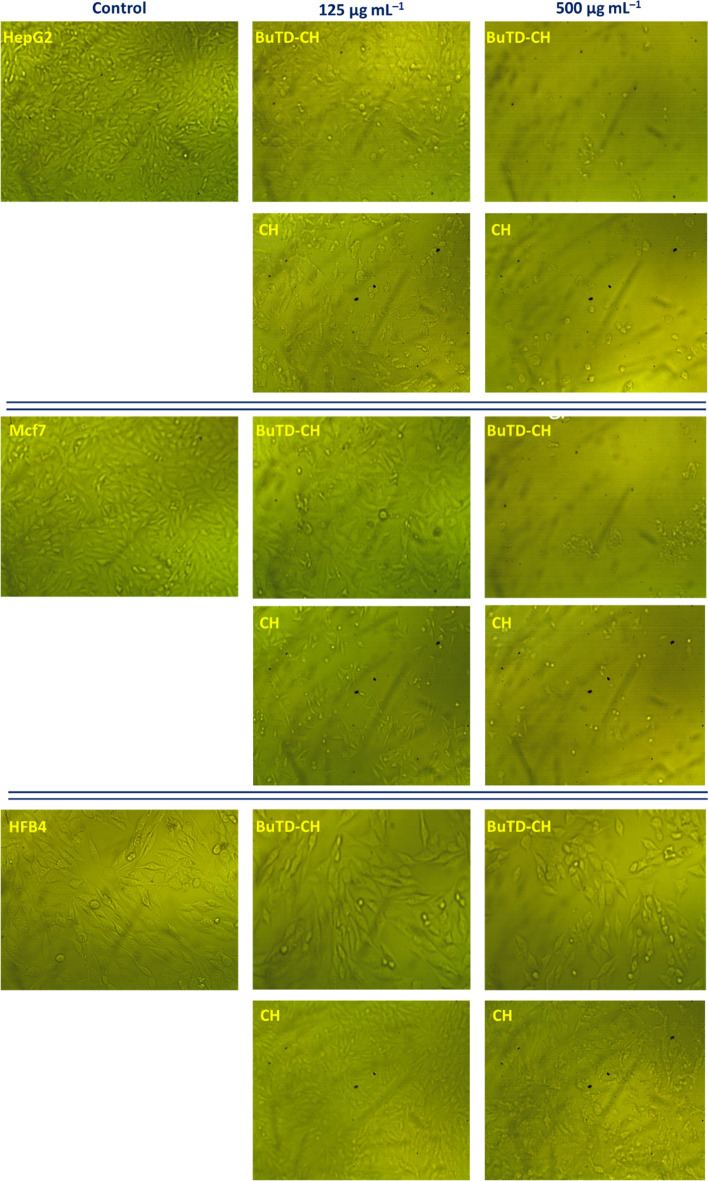
Microscope images of cancerous cell lines (HepG2 and MCF-7) and normal fibroblast. cells (HFB4) with and without BuTD-CH at different concentrations (0, 125, and 500 μg/ml).

**Figure 8:**
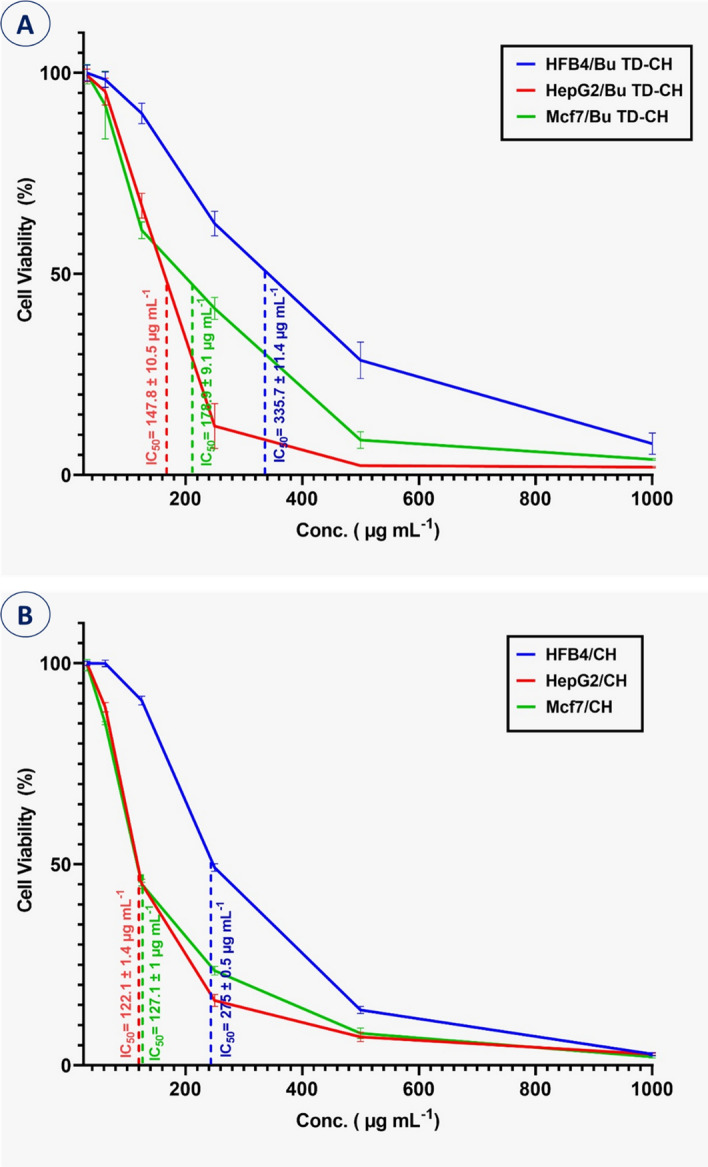
The cell viability assay using the MTT method of cancerous cell lines (HepG2, Mcf7) and normal fibroblast cells (HFB4) due to treatment with different concentrations of BuTD-CH (**A**) and unmodified chitosan (**B**).

Herein, the cytotoxicity of the newly modified chitosan compared to the unmodified was evaluated against two cancer cell lines (MCF-7, HepG2) and the non-carcinogenic (HFB4) cloned cell line by the MTT assay, while changes in the cellular phenotypic models were visualized by direct microscopy. The microscopic examination confirmed the efficacy of the BuTD-CH and unmodified compound against cancerous cells, which had undergone some morphological changes such as roundness, shrinkage, granulation, and migration, ending with the loss of the mono-layer characteristic of epithelial cells (Fig. 7). In the current study, six different concentrations of the modified chitosan (31.25–1000 μg ml^–1^) were attended for cytotoxicity investigations. The results revealed a dramatic reduction in the cell proliferation rate with increasing the synthesized chitosan concentration in a dose-dependent mode. We recorded analog phenotypic changes in both cancerous and non-cancerous cells treated with the modified chitosan. The BuTD-CH concentrations required for 50% of cell population mortality (IC_50_) were calculated from the constructed curve (Fig. 8). Interestingly, the IC_50_ for normal fibroblast cells = 335.7 ± 11.4 and 275 ± 0.5 μg ml^–1^ for modified and nonmodified chitosan respectively. These values for normal cells represent about twice the values specified for the two cancerous cells MCF-7 and HepG2 which were (178.9 ± 9.1 and 147.8 ± 10.5 μg ml^–1^, respectively) for functionalized BuTD–CH (Fig. 8A) and (127.1 ± 1.0 and 122.1 ± 1.4 μg ml^–1^, respectively) for unmodified chitosan (Fig. 8B). Since low concentrations of the BuTD-CH and chitosan without any modification manifested antiproliferative impact on the cancerous cells (MCF-7 and HepG2), while it can affect the population and survival of the normal fibroblasts (HFB4) only if applied in elevated concentrations, it is possible to exploit this target-orientation to create a therapeutic window for applying the synthesized BuTD-CH derivative as a chemotherapeutic agent. Thus, up to 180 μg ml^–1^ BuTD-CH could be safely used as a pharmacological agent for hepatocellular and adenocarcinoma therapy.

In the same context, the dose-dependent cytotoxicity of chitin and chitosan derivatives was proved against human liver cancer (HepG2) and rhabdomyosarcoma cell lines^73^. Recently, Resmi and coworkers reported the antiproliferative potential of the biogenic chitosan against adenocarcinoma (breast cancer cells MCF-7) in a concentration-dependent mode while being safe for the normal fibroblast cells (L929)^74^. The cytotoxicity of chitosan compounds could be attributed to the amino group that developed a positive charge in slightly acidic to neutral media (pKa∼6.5) enhancing chitosan water solubility and bio-adhesivity for promoted binding and penetration out of negative charge surfaces as basement and mucosal membrane^75^.

## Experimental protocols

### Materials

Chitosan (CH), Hydrazine carbothioamide (99%), and Butyl iodide (99%) were obtained from Sigma–Aldrich. Thionyl dichloride (99%) and carbon bisulfide (99%) were got from Alfa Aesar. Solvents (Methylene dichloride (HPLC) and Toluene (HPLC)), hydrochloric acid (37%), and sodium carbonate (99%) were purchased from Thermo Fisher Scientific and Nasr companies. Distilled water was prepared in a research laboratory. Succinic anhydride (CA) was synthesized as previously reported^76^.

### Determination of the molecular weight of chitosan

The average molecular weight of chitosan was determined by the Viscometry method using the Mark–Houwink equation as reported by Wang et al.^77^. Dried chitosan sample (0.1 g) was dissolved in 100 ml solvent (0.1 M sodium acetate + 0.2 M acetic acid). Five concentrations of chitosan solutions were prepared by dilution using a fresh solvent. The measurements were done at 30 °C using a Ubbelohde viscometer. The time flow of pure solvent and each chitosan concentration was conducted three times and the average was taken. For each concentration, the relative viscosity, specific viscosity, reduced viscosity, and inherent viscosity were calculated. The intrinsic [η] was obtained by extrapolating the reduced viscosity or inherent viscosity versus concentration data. Then, the molecular weight was calculated by the equations reported by Wang et al. It was found that the estimated viscosity's average molecular weight was 61 × 10^3^ g/mol.

### Deacetylation degree of chitosan

The deacetylation degree (DD) of chitosan was calculated based on its elemental analysis using Eq. (2) ^18^

2 $$DD\% { } = { }\frac{{{\text{n}}_{1} {\text{M}}_{{\text{c}}} - {\text{ M}}_{{\text{n}}} { }({\text{C}}/{\text{N}})_{{{\text{CH}}}} { }}}{{{\text{n}}_{2} {\text{M}}_{{\text{c}}} }} \times 100$$

where n_1_ and n_2_ are the number of carbon atoms in the chitin and acetamido groups, respectively. M_c_ and M_n_ are the molar mass of carbon and nitrogen, M_c_ = 12 and M_n_ = 14, respectively. From the calculations, DD was found to be 70.89%.

### Chemistry

#### Synthesis of 5-amino-1,3,4-thiadiazole-2-thiol. [TD-NH_2_]

The compound (TD-NH_2_) was obtained according to the experimental procedure in the method reported in our previous work^45^.

#### General procedure for the synthesis of [BuTD-NH_2_]

Two grams (15 mmol) of TD-NH_2_ were placed portion-wise into a solution of KOH (30 mmol) in ethyl alcohol (15 ml). The substance was stirred until it was completely dissolved in the reaction mixture. The conical flask was cooled in ice water and a halogenated saturated aliphatic hydrocarbon (butyl iodide) (15 mmol) was added over 50 min with vigorous stirring. After allowing the mixture to come to room temperature, it was stirred for around 8 h at room temperature. After completion of the reaction, the resulting solids were collected by filtration, washed with distilled H_2_O (3 × 20 ml), and recrystallized from MeOH/H_2_O to yield the title compound (2.66 g, 93.66%, pale-yellow, melting point: 116–118 °C)^78,79^.

#### Preparation of 4-((5-(butylthio)-1,3,4-thiadiazol-2-yl)amino)-4-oxo butanoic acid. [BuTD-COOH]

5-(butylthio)-1,3,4-thiadiazol-2-amine (10 mmol) and succinic anhydride (13.2 mmol) were mixed at room temperature in dry benzene in an Erlenmeyer flask fitted with a mechanical stirrer, and the mixture was stirred until full conversion was achieved. This was achieved after approximately 3 h. The product separates as a solid, and the solvent eventually evaporates under a vacuum. The residue was rinsed with distilled water 3 times and purified by recrystallization from ethanol/benzene to generate the target compound. (The yield is 2.1 g. (84%) of a white product melting at 242–244 °C).

#### The reaction of polysaccharide (chitosan) with 4-((5-(butylthio)-1,3,4-thiadiazol-2-yl)amino)-4-oxo butanoic acid. [BuTD-CH]

**Figure 9:**
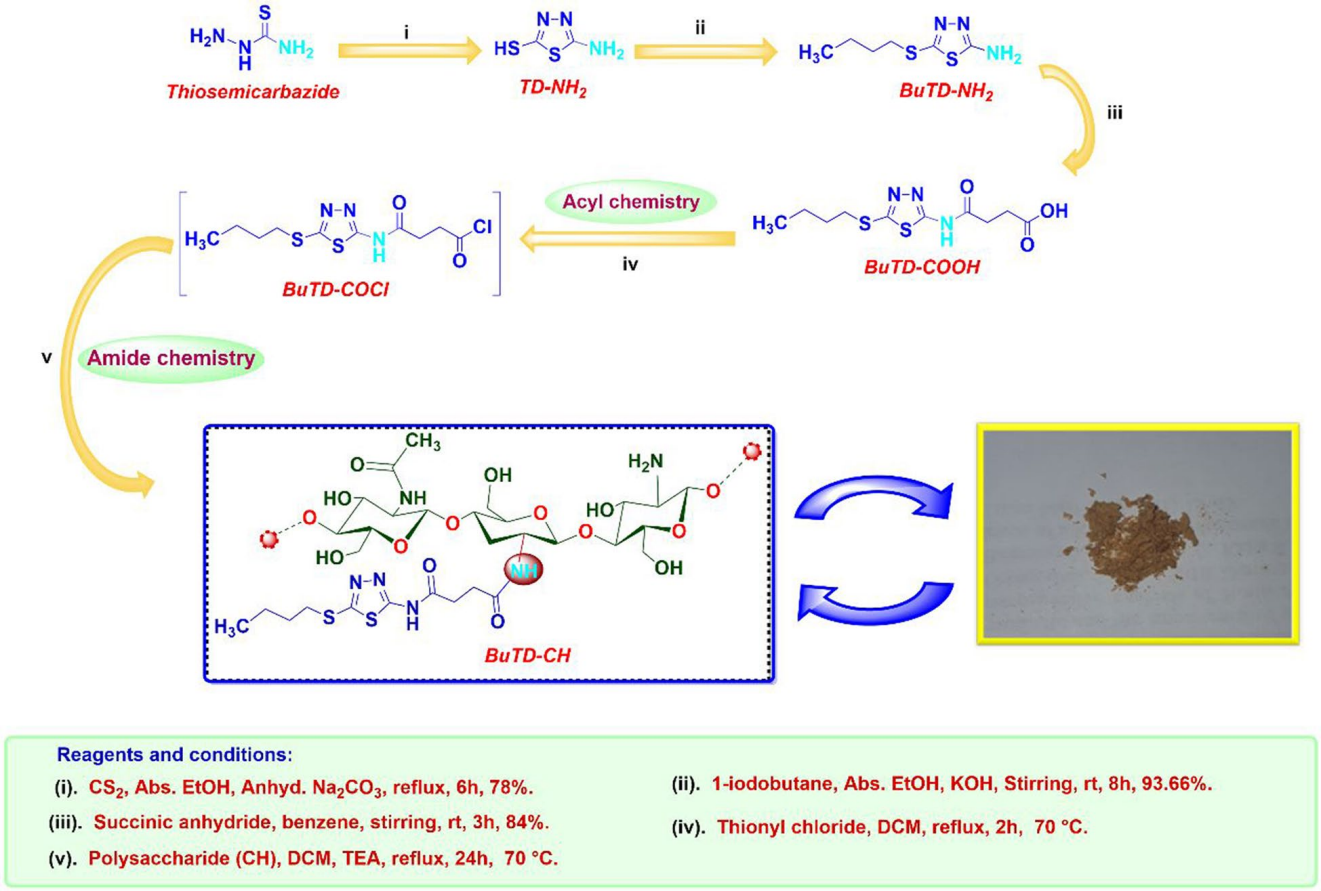
Schematic pathway for the synthesis of butylated thiadiazol chitosan derivative (BuTD-CH).

The modified chitosan, BuTD-CH, was synthesized by the formation of amide linkages. A solution of BuTD-COOH (6.57 mmol) in anhydrous dichloromethane was charged into a 250 ml two-necked round bottom flask equipped with a mechanical stirrer, a dropping funnel (50 ml), and an internal thermometer, and the solution was chilled to 0 °C in an ice/NaCl bath. The SOCl_2_ (0.13 mmol) in dry dichloromethane was added gradually to the flask via the dropping funnel at such a rate to keep the internal temperature below 5 °C. Then, the mixture was stirred under reflux for 2 h at 70 °C, and the solvent was extracted under reduced pressure, yielding a crude product ready for the following step without further purification. Chitosan powder (1.45 g) was added to a crude product in (50 ml) of anhydrous CH_2_Cl_2_, in the presence of a catalytic amount of triethyl amine (9.85 mmol). The reaction mixture was stirred under reflux for 24 h at 70 °C. Afterward, the solvent was evaporated under a vacuum. The product was washed with dichloromethane to get thiadiazole-grafted chitosan as demonstrated in Fig. 9.

### Instrumentation

The melting points of solid compounds were determined on the SMP50 Digital APP Apparatus (Bibby Scientific, Staffordshire, UK) 120/230 V instrument. Infrared spectra were measured on a Shimadzu FT-IR Affinity-1 Spectrometer, Infrared spectrometer the Nicolet iS10FT IR Spectrometer, Thermo Fisher Scientific Resolution 16, over a scanning range of 4000–400 cm^−1^, Band positions were described as wavenumbers at (ν cm^−1^) scale on the KBr plates at Faculty of Science-Ain Shams University, Cairo, Egypt. Proton/carbon-nuclear magnetic resonance (^1^H/^13^C-NMR, 500/125 MHz) spectra were carried out with JNM-ECA 500 II made by JEOL-JAPAN instrument and referenced to DMSO-d_6_ ((CD_3_)_2_SO) as a solvent and TMS as an internal reference. Chemical shifts are expressed in parts per million. In the NMR tabulation, s: singlet; d, doublet; t, triplet; q, quartet; m, multiplet; and br, broad peak. GC–MS was performed with Shimadzu Japan's GC–2010. Thermogravimetric analysis (TGA) of the sample was performed under N_2_ flow with heating rates measured at 10 °C/min from 0 to 500 °C on the Discovery SDT 650-Simultaneous DSC-TGA/DTA Instruments, USA. The sample was also analyzed for C, H, N, and S at the Microanalytical Center, Cairo University, Giza, Egypt, using the Elemental C–H–N–S AnalyzerVario El M, Germany, and values were obtained within ± 0.4% of the calculated values. XPS was collected on K-ALPHA (Thermo Fisher Scientific, USA) with monochromatic x-ray Al K-Alpha radiation from −10 to 1350 e.v. Spot size 400 micro m at pressure 10–9 mbar with full spectrum pass energy 200 e.v. and at narrow spectrum 50 e.v.

### Antimicrobial activity

The agar well diffusion assay was employed to explore the antimicrobial properties of modified and nonmodified chitosan against a group of clinical pathogens including Gram-negative bacteria (*Escherichia coli* ATCC8739 and *Pseudomonas aeruginosa* ATCC9022), Gram-positive bacteria (*Bacillus subtilis* ATCC6633 and *Staphylococcus aureus* ATCC6538), and the model of unicellular fungi (*Candida albicans* ATCC10231). *Candida albicans* and the tested bacterial strains were cultured in yeast extract peptone dextrose (YEPD) broth media and nutrient broth for one day at 35 ± 2 °C^80^. In each experiment, 50 μl of each microbial progeny (O.D = 1.0) were seeded onto 100 ml of sterilized Muller Hinton agar media (Oxoid) and dispensed in Petri dishes. 0.7 mm wells were cut in the solidified seeded plates. A stock solution of the chitosan (modified and nonmodified) was prepared in DMSO (300 μg/1.0 ml DMSO) and made ready for double-fold concentrations (200, 100, 50, 25, and 12.5 μg ml^–1^) that were used to determine the value of the minimum inhibitory concentration (MIC). Finally, 100 μL of each chitosan concentration was decanted into an agar well, along with 100 μl of pure DMSO as a negative control and 100 μl of penicillin G, ciprofloxacin, and ketoconazole as positive controls for Gram-positive, Gram-negative bacteria, and unicellular fungi, respectively. The laden Muller Hinton agar plates were refrigerated for 60 min before being incubated at 35 ± 2 °C for one day^81^. The activity of the butylated chitosan and MIC values were determined by measuring the zone of inhibition (ZOI) that was appointed using standard deviation (± SD) in three independent repetitions.

### In-vitro cytotoxicity assay

#### Cell lines culture

The MCF-7 (adenocarcinoma), HepG2 (human liver cancer), and HFB4 (normal melanocytes) cell lines were purchased from the Holding Company for Biological Products and Vaccines (VACSERA) Cairo, Egypt.

#### Compound stock preparation

1 mg of tested compounds (modified and unmodified chitosan) was solved in 1 ml of Gibco Roswell Park Memorial Institute (RPMI) medium and sterilized by filtering through a 0.22 μm syringe filter (Millipore).

#### MTT assay

The cytotoxicity of modified chitosan against cancer (MCF-7 and HepG2) and normal (HFB4) cell lines compared to the unmodified compound was evaluated by conducting a Dimethyl thiazolyl tetrazolium bromide (MTT) assay. The tested cells were grown in 96-well tissue culture plates (100 μl/well, 1 × 10^5^ cells) and incubated at 37 °C in a humidified condition in a 5% CO_2_ incubator for 24 h. After a confluent sheet of cells was formed, the cell monolayer was washed twice with washing media and incubated for 48 h in maintenance media (RPMI medium with 2% serum) treated with double-fold dilutions (1000–31.25 μg ml^–1^) of tested chitosan (modified or unmodified), with three wells receiving only RPMI medium as control. After incubation, culture media were decanted and 50 μL of fresh MTT solution (5 mg/mL in PBS, BIO BASIC CANADA INC) were added to each well, and thoroughly mixed for 5 min on a shaking table (150 rpm), Plates were incubated for 4 h to allow metabolization of MTT. After incubation, the media were dumped off and the developed formazan crystals were dissolved in DMSO (10%), and the plates were shaken in the dark for 30 min. Finally, the optical density was measured at 570 nm in a multi-well ELISA plate reader^82,83^. Changes in cell morphology were visualized by a phase contrast microscope. Cell viability was calculated by the following equation (Eq. 3)^84^:

3 $${\text{Cell viability}}\;(\% ) = \frac{\text{Absorbance of treated sample}}{{\text{Absorbance of control}}} \times 100$$

### Statistical analysis

Data of biological activities are represented as the means of three independent replicates. The collected data were analyzed using the statistical package SPSS v17. The mean difference comparison between the treatments was analyzed by t-test or the analysis of variance (ANOVA) and subsequently by the Tukey HSD test at *p* < 0.05.

## Conclusions

In this study, a new thiadiazole chitosan derivative was formed by the reaction of a new synthesized compound, 4-((5-(butylthio)-1,3,4-thiadiazol-2-yl) amino)-4-oxo butanoic acid with chitosan. The obtained modified chitosan was characterized using FT-IR, ^1^H/^13^C-NMR, GC–MS, TGA, elemental analysis, and XPS. The synthesized chitosan derivatives showed high antimicrobial and in-vitro cytotoxicity activity. The antimicrobial investigation indicated that the lowest MIC of the chitosan derivative (BuTD-CH) was 25 μg ml^–1^ against *B. subtilis*, *E. coli,* and *C. albicans* with ZOIs of 10.38 ± 0.76, 10.33 ± 0.57, and 9.66 ± 0.57 mm, respectively. Whereas this value was increased to 50 μg ml^–1^ against *S. aureus* and *P. aeruginosa* with ZOIs of 10. 66 ± 0.28 and 10.33 ± 0.57 mm, respectively. The modified chitosan showed high antimicrobial activity compared to the unmodified compound. The synthesized chitosan derivative showed high efficacy against breast cancer cell lines (MCF-7) and human liver cancer cell lines (HepG2) and caused a dramatic reduction in the cell proliferation rate with increasing its concentration. The low concentrations of the chitosan derivative (BuTD-CH) and chitosan manifested an antiproliferative impact on the cancerous cells (MCF-7 and HepG2), while it could affect the population and survival of the normal fibroblasts (HFB4) only if applied in elevated concentrations. It is possible to exploit this target orientation to create a therapeutic window for applying chitosan compound as a chemotherapeutic agent. The obtained data confirmed the main hypothesis of the current study, which was the possibility of integration of functionalized chitosan with new thiadiazole derivatives in biomedical applications.

## Supplementary Information

Supplementary Information.

## Data Availability

The datasets used and/or analyzed during the current study are available from the corresponding author on reasonable request.
