# Supplementary material for: New thiadiazole modified chitosan derivative to control the growth of human pathogenic microbes and cancer cell lines
Source: Sci Rep. 2022 Dec 11;12:21423. doi: 10.1038/s41598-022-25772-4 (PMC9742148; doi:10.1038/s41598-022-25772-4)
Supplement: Supplementary file 1 — Supplementary Information. [file 41598_2022_25772_MOESM1_ESM.docx]

**New thiadiazole modified chitosan derivative to control the growth of human pathogenic microbes and cancer cell lines**

Ahmed G. Ibrahim ^1^, Amr Fouda ^2,^ *, Walid E. Elgammal ^1^, Ahmed M. Eid ^2^, Mohamed M. Elsenety ^1^, Ahmad E. Mohamed ^1^, Saber M. Hassan ^1^

^1^ Department of Chemistry, Faculty of Science, Al-Azhar University, Nasr City, Cairo, Egypt

^2^ Department of Botany and Microbiology, Faculty of Science, Al-Azhar University, Nasr City, Cairo, Egypt.

*Corresponding author: Amr Fouda

E-mail address: [amr_fh83@azhar.edu.eg](mailto:amr_fh83@azhar.edu.eg)


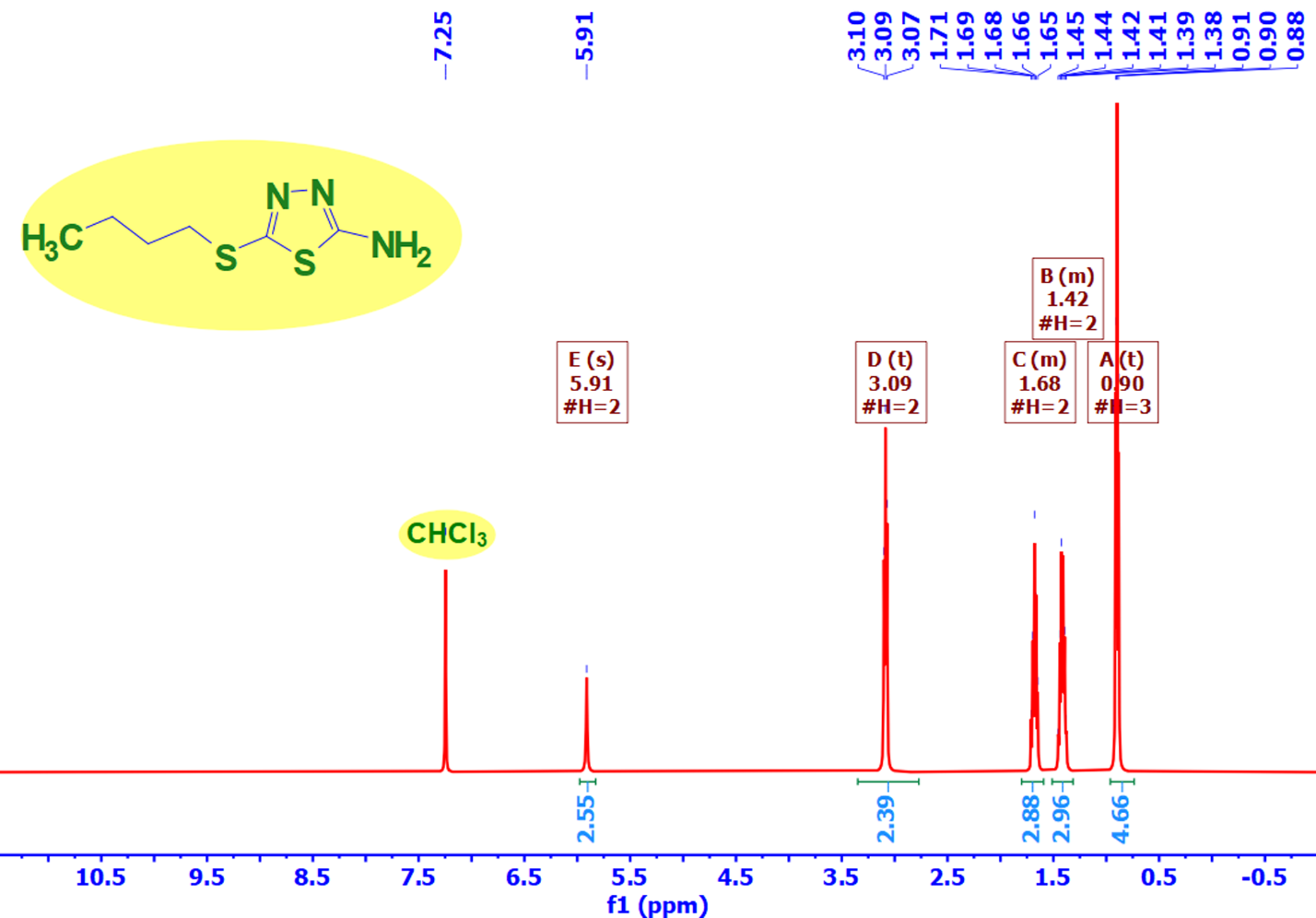


**Figure S1a.** ^1^H-NMR (CDCl_3_) analysis of BuTD-NH_2_.


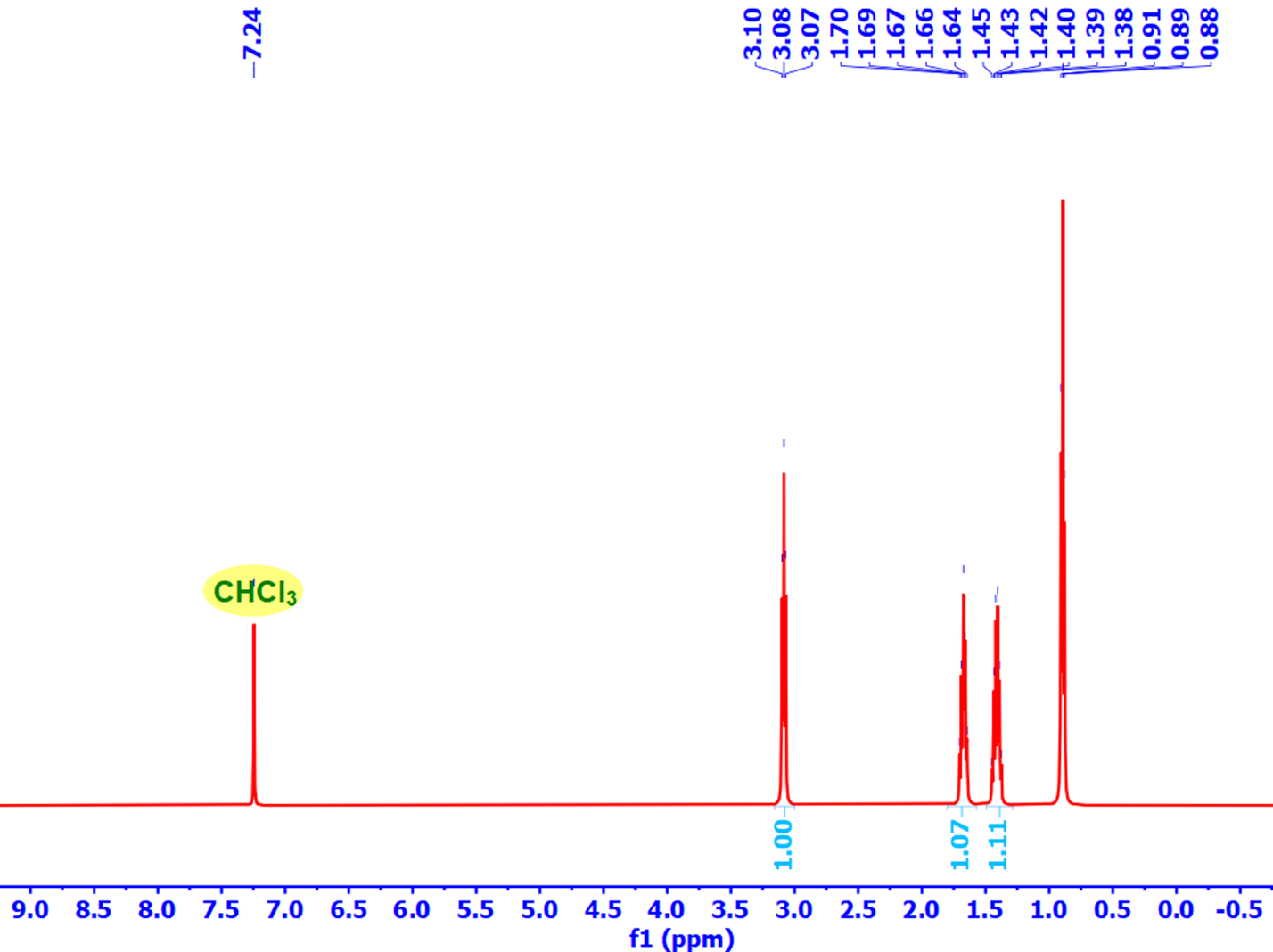


**Figure S1b.** ^1^H-NMR(CDCl_3_\D_2_O) analysis of BuTD-NH_2_.


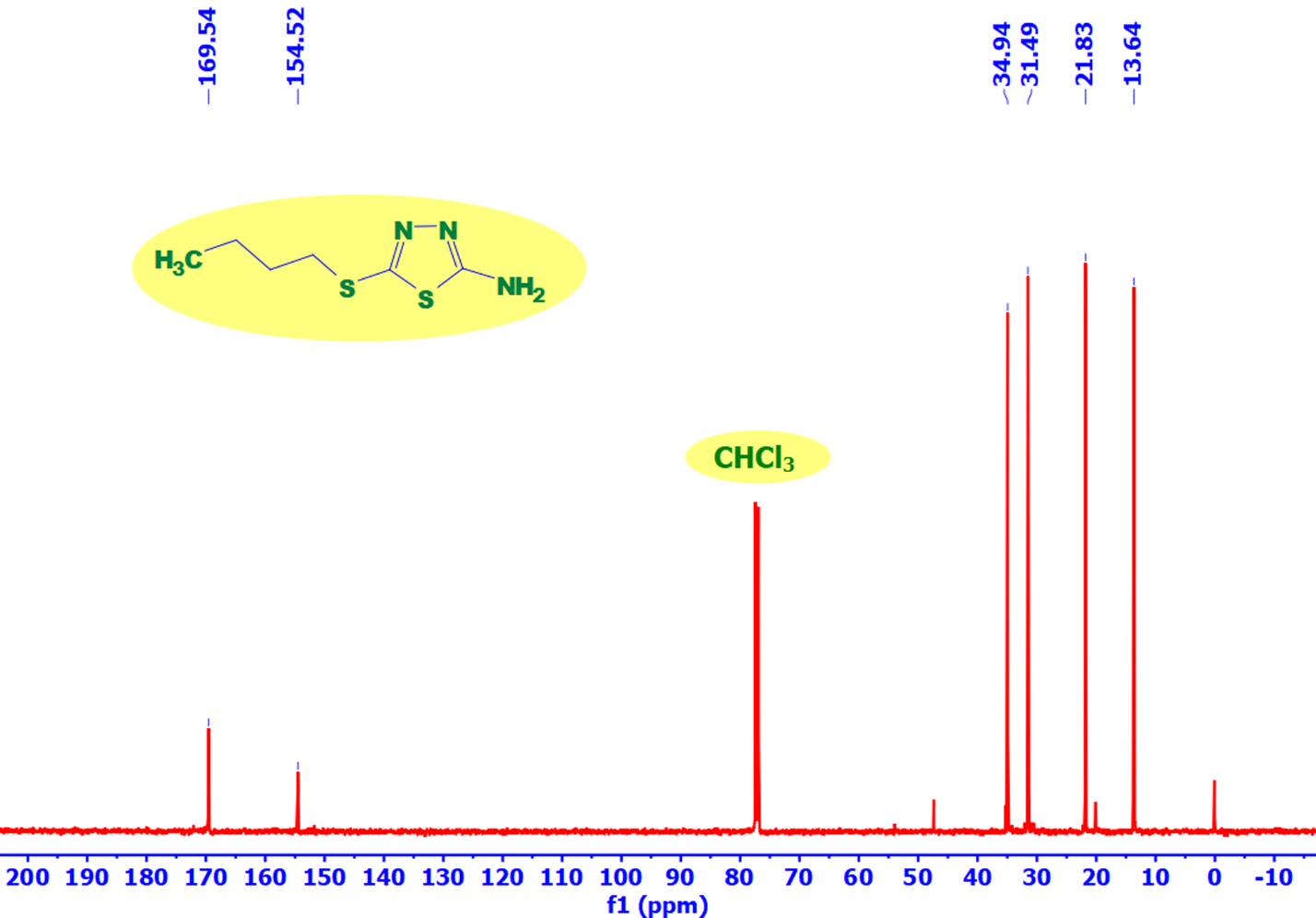


**Figure S1c.** ^13^C-NMR (CDCl_3_) analysis of BuTD-NH_2_.


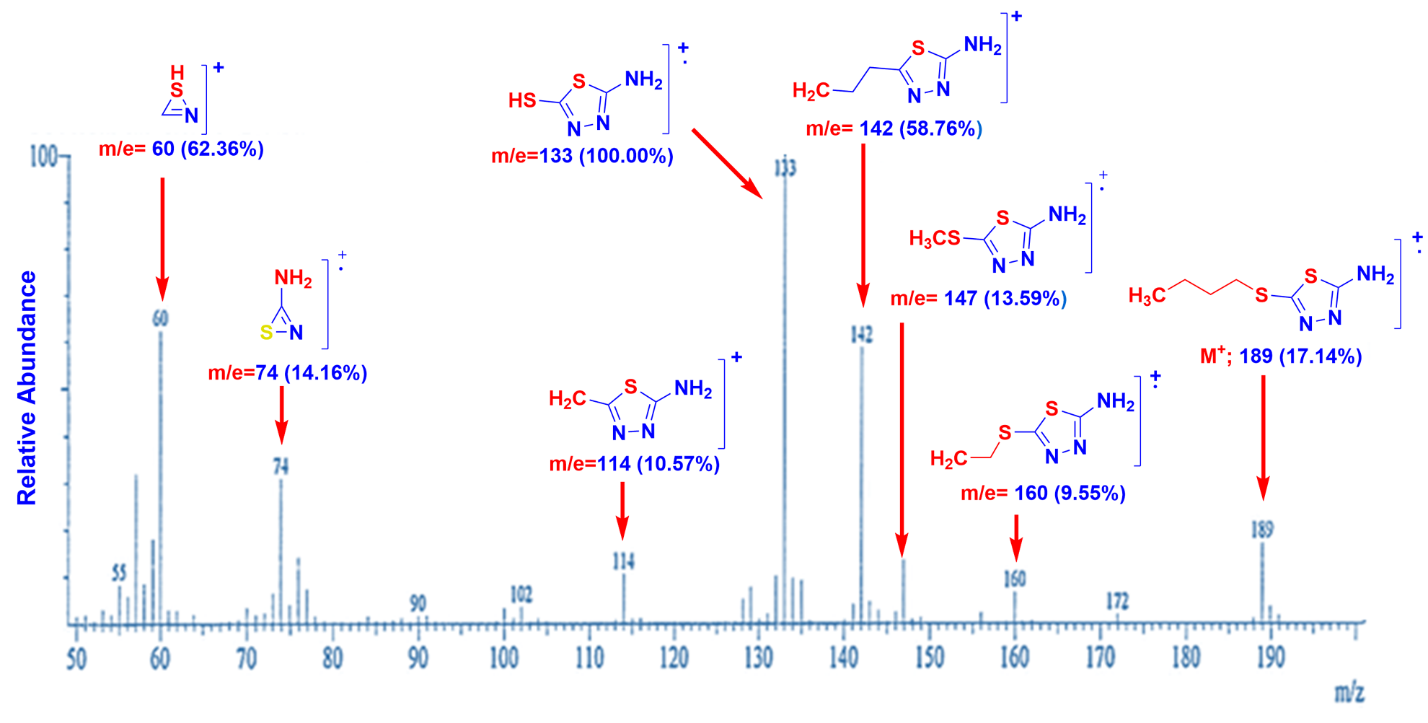


**Figure S1d.** Mass spectra analysis of BuTD-NH_2_.


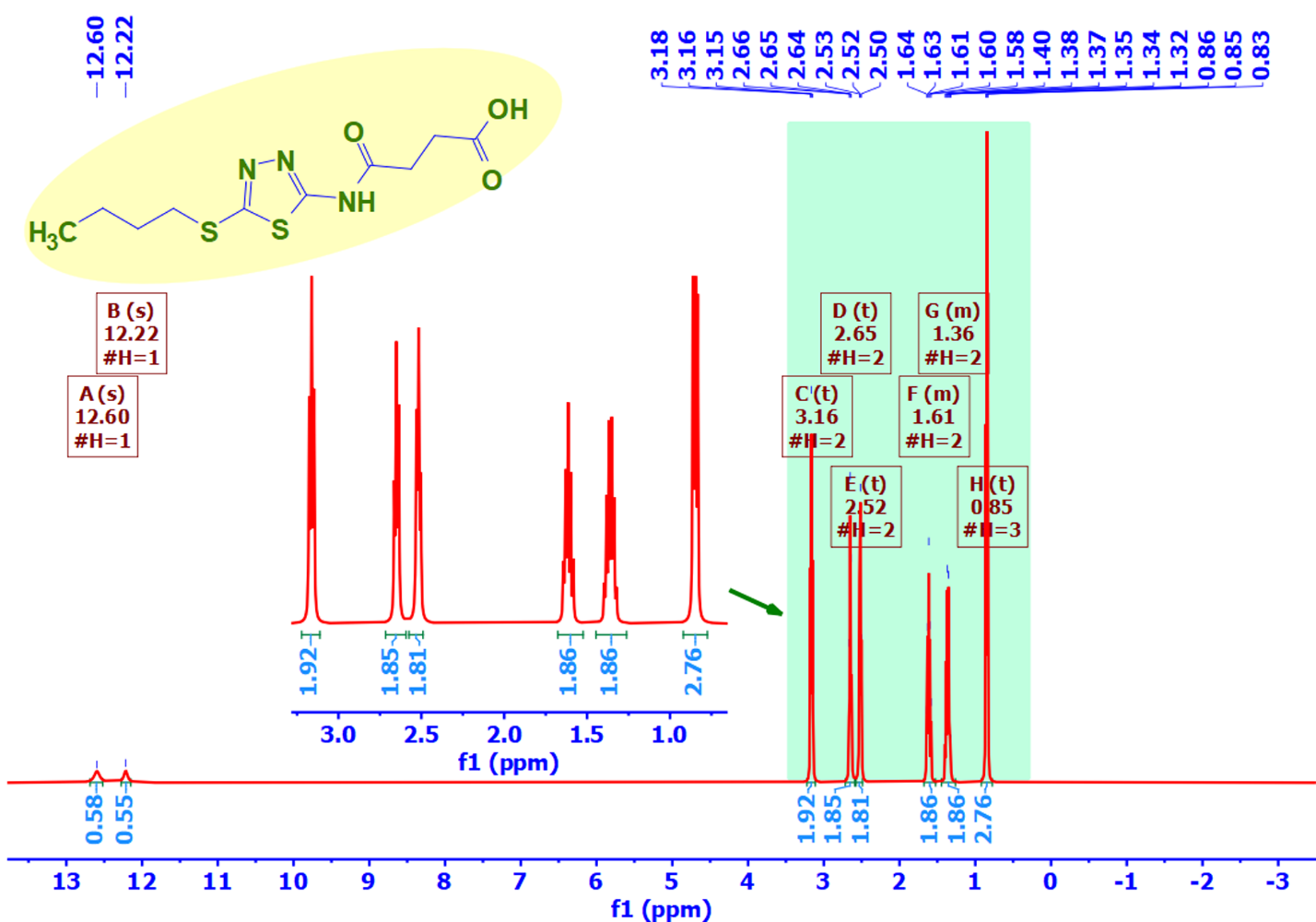


**Figure S2a.** ^1^H-NMR (DMSO-D_6_) analysis of BuTD-COOH.


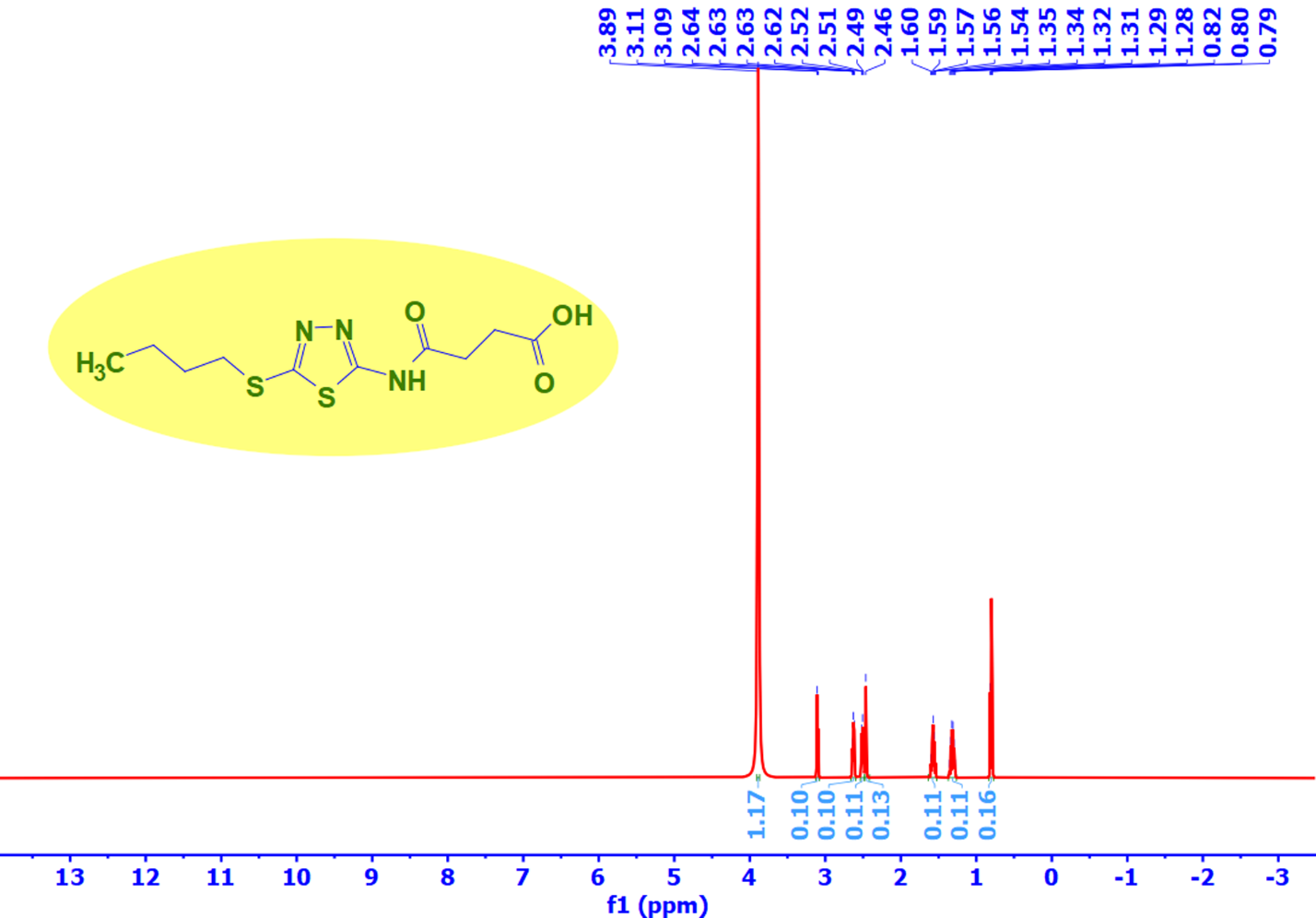


**Figure S2b.** ^1^H-NMR(DMSO-D_6_\D_2_O) analysis of BuTD-COOH.


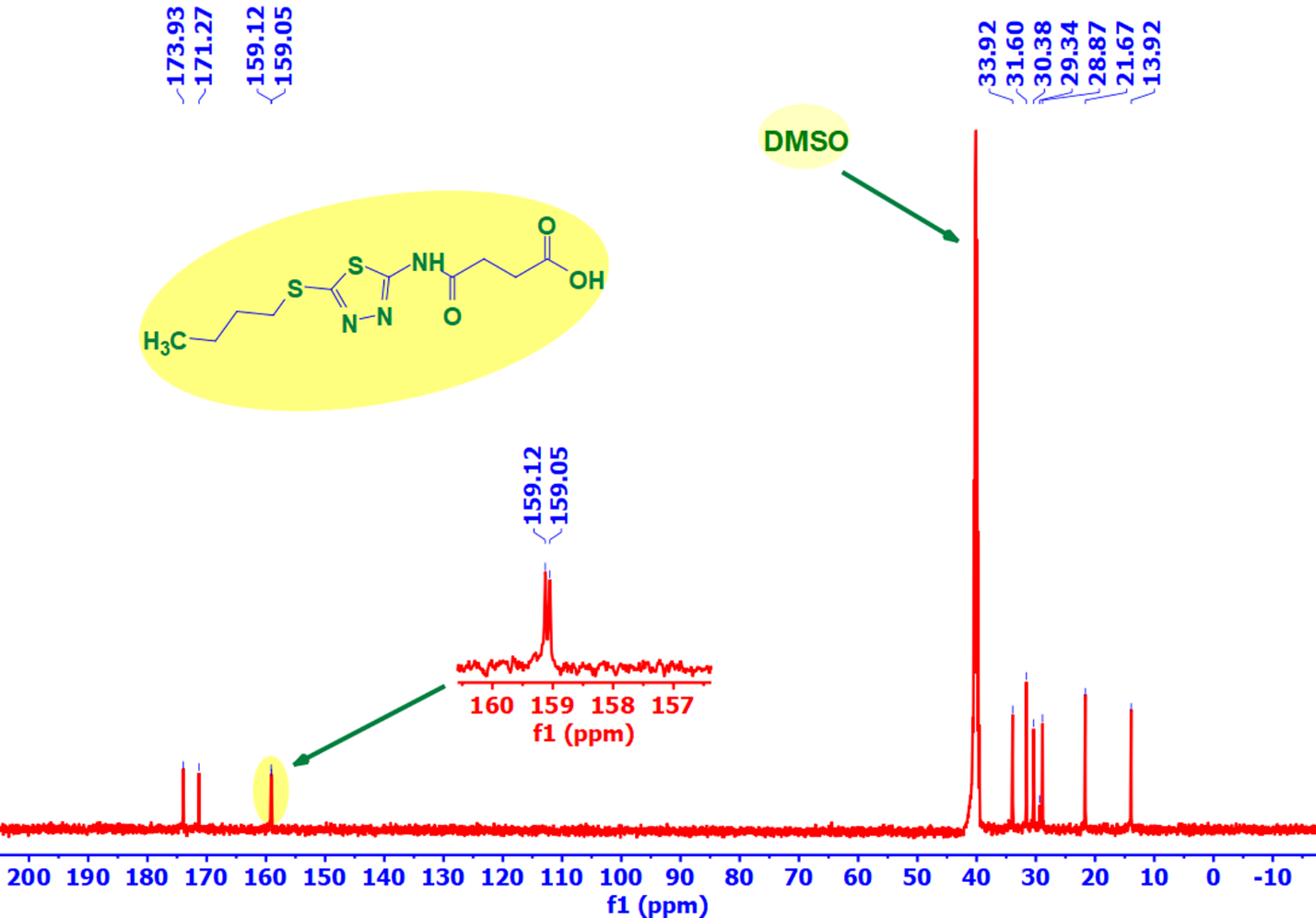


**Figure S2c.** ^13^C-NMR (DMSO-d_6_) analysis of BuTD-COOH.


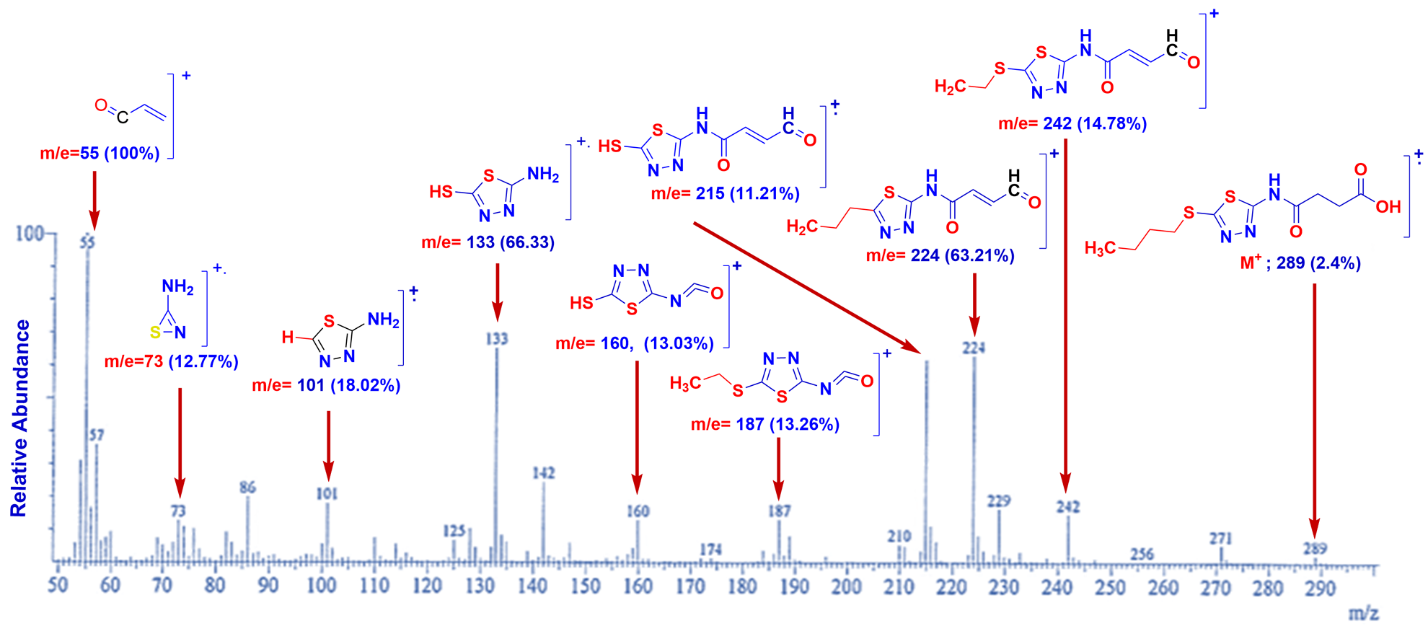


**Figure S2d.** The EI-mass spectra analysis of Bu-TD-COOH.
